# A novel natural killer cell-related signatures to predict prognosis and chemotherapy response of pancreatic cancer patients

**DOI:** 10.3389/fgene.2023.1100020

**Published:** 2023-03-23

**Authors:** Yongting Lan, Qing Jia, Min Feng, Peiqing Zhao, Min Zhu

**Affiliations:** ^1^ Department of Gastroenterology, Zibo Central Hospital, Zibo, China; ^2^ Department of Neonatology, Zibo Maternal and Child Health Hospital, Zibo, China

**Keywords:** natural killer cells, pancreatic cancer, consensus clustering, nomogram, methylation, programmed cell death, prognosis

## Abstract

**Background:** Natural killer (NK) cells are involved in monitoring and eliminating cancers. The purpose of this study was to develop a NK cell-related genes (NKGs) in pancreatic cancer (PC) and establish a novel prognostic signature for PC patients.

**Methods:** Omic data were downloaded from The Cancer Genome Atlas Program (TCGA), Gene Expression Omnibus (GEO), International Cancer Genome Consortium (ICGC), and used to generate NKG-based molecular subtypes and construct a prognostic signature of PC. NKGs were downloaded from the ImmPort database. The differences in prognosis, immunotherapy response, and drug sensitivity among subtypes were compared. 12 programmed cell death (PCD) patterns were acquired from previous study. A decision tree and nomogram model were constructed for the prognostic prediction of PC.

**Results:** Thirty-two prognostic NKGs were identified in PC patients, and were used to generate three clusters with distinct characteristics. PCD patterns were more likely to occur at C1 or C3. Four prognostic DEGs, including MET, EMP1, MYEOV, and NGFR, were found among the clusters and applied to construct a risk signature in TCGA dataset, which was successfully validated in PACA-CA and GSE57495 cohorts. The four gene expressions were negatively correlated with methylation level. PC patients were divided into high and low risk groups, which exerts significantly different prognosis, clinicopathological features, immune infiltration, immunotherapy response and drug sensitivity. Age, N stage, and the risk signature were identified as independent factors of PC prognosis. Low group was more easily to happened on PCD. A decision tree and nomogram model were successfully built for the prognosis prediction of PC patients. ROC curves and DCA curves demonstrated the favorable and robust predictive capability of the nomogram model.

**Conclusion:** We characterized NKGs-derived molecular subtypes of PC patients, and established favorable prognostic models for the prediction of PC prognosis, which may serve as a potential tool for prognosis prediction and making personalized treatment in PC.

## 1 Introduction

Pancreatic cancer (PC) as a lethal malignancy shows a high mortality worldwide, causing over 331000 deaths per year globally (Rawla et al., 2019). Although advances in the treatment of PC, patients who received surgical resection have a five-year survival rate ranging from 10% to 25% (Siegel et al., 2020). PC was usually diagnosed at a late stage due to the impalpable symptoms at the early stage, and approximately 80%–85% of PC was unresectable or metastatic at the time of diagnosis (Okasha et al., 2017). Currently, chemotherapy is the main treatment for PC but remains an unsatisfactory prognosis, and more effective and precise therapies are required (Mizrahi et al., 2020).

Immunotherapy has been recently developed to help improve the prognosis of various cancer types, such as renal cell carcinoma (Cho et al., 2017), non-small cell lung cancer (Hellmann et al., 2018), hematologic malignancies (Nelson and Paulos, 2015), and melanoma (Ribas and Wolchok, 2018). The principle of tumor immunotherapy is to fight against tumors through the activation of immune system, during which restarting and maintaining tumor-immune cycle plays a crucial role. Therapeutic targeting of immune checkpoints with immune checkpoint inhibitors has revolutionized cancer treatment (Komatsubara and Carvajal, 2017; Pulluri et al., 2017; Considine and Hurwitz, 2019). It was reported that checkpoint blockade in combination with GVAX has the potential for clinical benefit for patients with PC (Le et al., 2013). T-cell immunity is associated with the exceptional outcome of the few long-term survivors. A study identified unique neoantigens as T-cell targets in PC patients, which might be used to guide the application of immunotherapies (Balachandran et al., 2017). Pembrolizumab is a PD-1 inhibitor and has been approved for tumor patients with deficient mismatch repair or high microsatellite instability, including PC (Boyiadzis et al., 2018). However, the efficacy was restricted to a rare population due to the complex, highly immunosuppressive tumor microenvironment of PC (O'Reilly et al., 2019).

The tumor immune microenvironment (TME) contains tumor cells, immune cells, cytokines, etc., and its heterogeneity can potentially impact the patient’s response to immunotherapy. Natural killer (NK) cells are a subset of innate immune cells and play a crucial role as effector cells against tumors. NK cell can directly kill malignant even at a relatively low ratio in the early presence of tumors (Huntington et al., 2007) or promotes adaptive T-cell immunological responses to limit cancer cell aggressiveness (López-Soto et al., 2017). The activation of NK cells is controlled by the integration of signals from cytokine receptors and a range of germline-encoded inhibitory and activating receptors (Moretta et al., 2006; Lanier, 2008). Studies found that NK cell activity was significantly negatively correlated with the risk of malignancy (Imai et al., 2000), and patients with a higher NK cell infiltration into cancers had better outcomes (Coca et al., 1997; Ishigami et al., 2000; Cursons et al., 2019). Cutting-edge immunotherapy targeting NK cells exerts great potential in the treatment of cancer and become an attractive alternative to T cell immunotherapies (Guillerey et al., 2016; Souza-Fonseca-Guimaraes et al., 2019). Accumulating evidence described the molecular characteristics of NK cells in various cancers (Sun et al., 2021a; Sun et al., 2021b), but a comprehensive molecular characterization of NK cells in PC remains poorly understood.

In the present study, the PC patients were clustered on the basis of natural killer cell-related genes (NKGs), and further comparison of the clinicopathological, mutational, immunological and pathway characteristics among subtypes was conducted. In addition, we identified prognostic differentially expressed genes (DEGs) among subgroups and constructed a risk signature for prognosis prediction. The decision tree and nomogram model were built using clinicopathological features and the risk signature to assist in prognostic prediction and personalized treatment of patients with PC.

## 2 Materials and methods

### 2.1 Data collection and preprocessing

Transcriptome files and clinicopathological data of patients with PC were obtained from the Cancer Genome Atlas Program (TCGA) (https://tcga-data.nci.nih.gov/tcga/), Gene Expression Omnibus (GEO) (https://www.ncbi.nlm.nih.gov/geo/), and the International Cancer Genome Consortium (ICGC) (https://www.icgc.org) databases. After removal of patients without complete clinical information and outcome status, as well as follow-up of fewer than 30 days, 176 PC patients from the TCGA pancreatic adenocarcinoma (TCGA-PAAD) cohort were retained as a training set. Ensembl was converted into gene symbol, and median value was kept when a genes had multiple gene symbols. The validation set contains 63 samples from the GSE57495 cohort and 215 patients of the PACA-CA cohort from the ICGC database. When multiple gene symbols appear or multiple probes appear for a gene, the median is taken as the gene expression value. A total of 134 human NKGs were downloaded from the ImmPort (https://www.immport.org/resource) database.

### 2.2 Consensus clustering

The prognostic NKGs were identified *via* univariate Cox regression analysis and were used to perform consensus clustering of PC patients. Consensus clustering analysis was conducted using the “ConsensusClusterPlus” R package to determine subgroups of PC patients based on the prognostic NKGs (Wilkerson and Hayes, 2010). The best classification was determined using the partition around medoids (PAM) algorithm and 1-Pearson correlation distance, with 500 bootstraps.

### 2.3 Risk score

The DEGs among NKGs-derived clusters were screened out using “limma” package according to the false discovery rate (FDR) < 0.05 and |log2 [fold change (FC)]| > log2 (2) (Ritchie et al., 2015). The univariate and the least absolute shrinkage and selection operator (LASSO) Cox regression analysis were adopted to identify and filter prognosis-related NKGs, respectively. Finally, by choosing the optimal penalty parameter lambda correlated with the minimum 10-fold cross-validation, multivariate Cox regression analysis was then implemented to establish the prognostic signature. The formula for the risk signature was as follows: risk score = ∑βi×Expi . Where the βi represents the coefficient and Expi represents the normalized expression level of a gene. Two risk groups (high and low) were generated by a threshold of zero, and K–M analysis was conducted to compare overall survival (OS) differences between the high- and low-risk groups. The receiver operating characteristic (ROC) analysis was performed to estimate the predictive accuracy of the risk score.

### 2.4 Gene set enrichment analysis

GSEA was performed to analyze the differences in specific gene sets using the “GSVA” R package (Hänzelmann et al., 2013). The hallmark gene sets from the Molecular Signatures Database (MSigDB), the inflammation-related gene sets (Liu et al., 2020), and the angiogenesis-related gene set (Masiero et al., 2013) were used to be analyzed. These pathways with the FDR <0.05 was considered to be significant. Functional enrichment analysis included Kyoto Encyclopedia of Genes and Genomes (KEGG) and Gene Ontology (GO) (biological process (BP), cellular component (CC), and molecular function (MF)) analysis was performed on DEGs in clusters using WebGestaltR package (Yu et al., 2012).

### 2.5 Immune infiltration, chemotherapeutic sensitivity, and immunotherapy response predictions

The relative proportion of immune cells was calculated using the CIBERSORT algorithm (https://cibersort.stanford.edu/), which performs cell type enrichment analysis from gene expression data for 22 immune cells. The “ESTIMATE” R package was applied to estimate and extrapolate the fraction of stromal and immune cells in tumor samples (Yoshihara et al., 2013). The expression levels of the immune checkpoints were compared in different groups. To predict the chemosensitivity of osteosarcoma patients to several common anti-cancer drugs (methotrexate, paclitaxel, cisplatin, and doxorubicin), we adopted the “pRRophetic” R package to infer the half-maximal inhibitory concentration (IC_50_) values by constructing the ridge regression model based on Genomics of Drug Sensitivity in Cancer (GDSC) (www.cancerrxgene.org/) cell line expression spectrum and gene expression profiles (Geeleher et al., 2014).

### 2.6 Establishment of a predictive nomogram

The decision tree model was applied to classify subgroups based on clinicopathogicial features and risk scores by using the “rpart” R package (https://cran.r-project.org/web/packages/rpart/index.html). The independent prognostic factors of OS for PC were identified by univariate and multivariate Cox regression analysis. A nomogram integrating the risk signature and independent prognostic clinicopathological factors was constructed in the TCGA cohort by the “rms” R package (https://cran.r-project.org/web/packages/rms/index.html). The calibration curves were utilized to evaluate the prediction accuracy between the predicted 1-, 2- and 3-year OS probabilities and the actual observations. The discriminate ability of the nomogram was assessed by time-dependent ROC curves. The decision curve analysis (DCA) was conducted to test the clinical utility of the nomogram using the “rmda” R package (https://cran.r-project.org/web/packages/rmda/index.html).

### 2.7 Mutation analysis

Tumor mutation burden (TMB) is was determined as the number of somatic indels and base substitutions per million bases in the coding region of the genome detected. Gene mutation data of PC patients were downloaded from the TCGA database and TMB was calculated using the “maftools” package (Mayakonda et al., 2018) as previously described (Chalmers et al., 2017).

### 2.8 Programmed cell death (PCD) analysis

12 PCD patterns were acquired from previous (Zou et al., 2022). Altogether, 580 apoptosis genes, 52 pyroptosis genes, 87 ferroptosis genes, 367 autophagy genes, 14 cuproptosis genes, 9 parthanatos genes, 15 entotic cell death genes, 101 necroptosis genes, 8 netotic cell death genes, 7 alkaliptosis genes, 220 lysosome-dependent cell death genes, and 5 oxeiptosis genes were collected. Based on the expression data of above gene sets, ssGSEA analysis was conducted on tumor samples using the R package GSVA.

### 2.9 Statistical analysis

The R software (v3.6.3) was used for statistical analyses. Wilcoxon test compared differences between two groups. Survival differences were compared using K–M curves with a Log-rank test. The Cox proportional hazard model was performed to estimate the β regression coefficient, hazard ratios, *p*-value, and their corresponding 95% confidence interval for each of the selected risk predictors. a nomogram was constructed with the “rms” package in R. The C-index and calibration curve with the bootstrap method were used to evaluate the prediction performance of the nomogram. A *p*-value <0.05 was deemed to be a statistical significance.

## 3 Results

### 3.1 Molecular subtypes derived from natural killer cell-related genes

**FIGURE 1:**
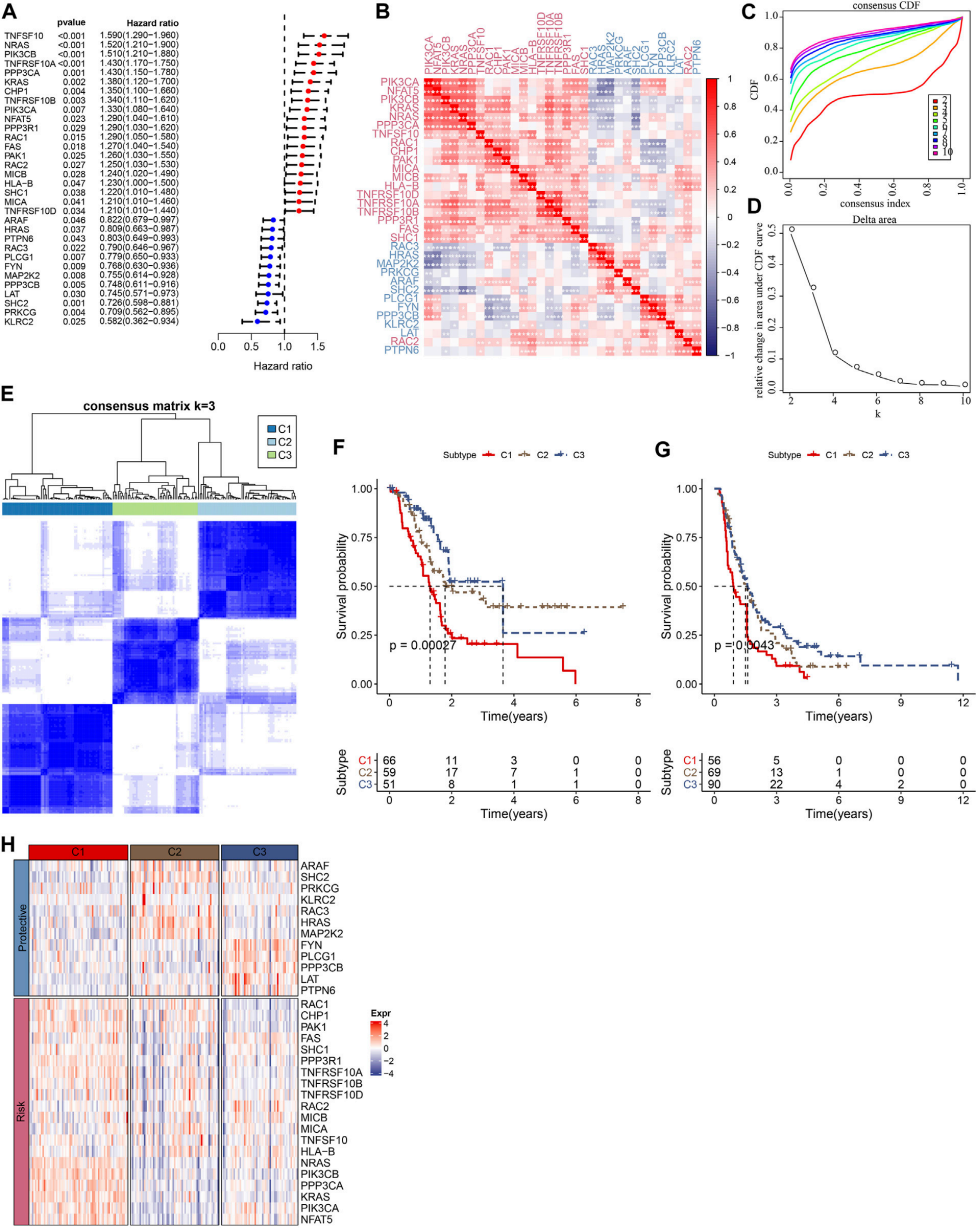
Consensus clustering of PC patients based on NKG signature. **(A)** Forest plot of prognosis-related NKGs in the TCGA-PAAD cohort; **(B)** The correlations among 32 prognosis-related NKGs in the TCGA-PAAD cohort; **(C)** Consensus cumulative distribution function (CDF) diagram when different k values, **(D)** Delta area plot for the relative change in the area under CDF curve for k compared to k-1, **(E)** Consensus matrix when the number of groups (k) = 3. In the consensus matrix, white meant that samples were impossibly clustered together, and dark blue meant that samples were always clustered together. Both rows and columns of the matrix represented samples, **(F)** and **(G)** represented the survival analysis of the clusters in the TCGA-PAAD cohort and PACA-CA cohort, respectively. **(H)** The heatmap of expression of 32 prognosis-related NKGs in the TCGA-PAAD cohort.

The flowchart is shown in Supplementary Figure S1. To obtain molecular subtypes of PC based on NKG, we first identified 32 NKGs that were significantly associated with the prognosis of PC (*p* < 0.05, Figure 1A). Notably, positive correlations among the expression of the 32 NKGs were observed in Figure 1B. Subsequently, consensus clustering of the 32 NKGs generated three stable clusters (C1, C2, and C3) in the TCGA-PAAD cohort (Figures 1C–E). Survival analysis demonstrated that the C3 cluster had a favorable prognosis whereas the C1 cluster had a poorer prognosis (Figure 1F). The individuals in the PACA-CA cohort were also divided into three clusters, which exerted similar prognosis characteristics as the clusters in the TCGA-PAAD cohort (Figure 1G). Among the 32 NKGs, the risk genes were generally overexpressed in the C1 cluster, and the protective genes were mainly elevated in the C3 clusters (Figure 1H).

### 3.2 Genomic landscapes among molecular subtypes

**FIGURE 2:**
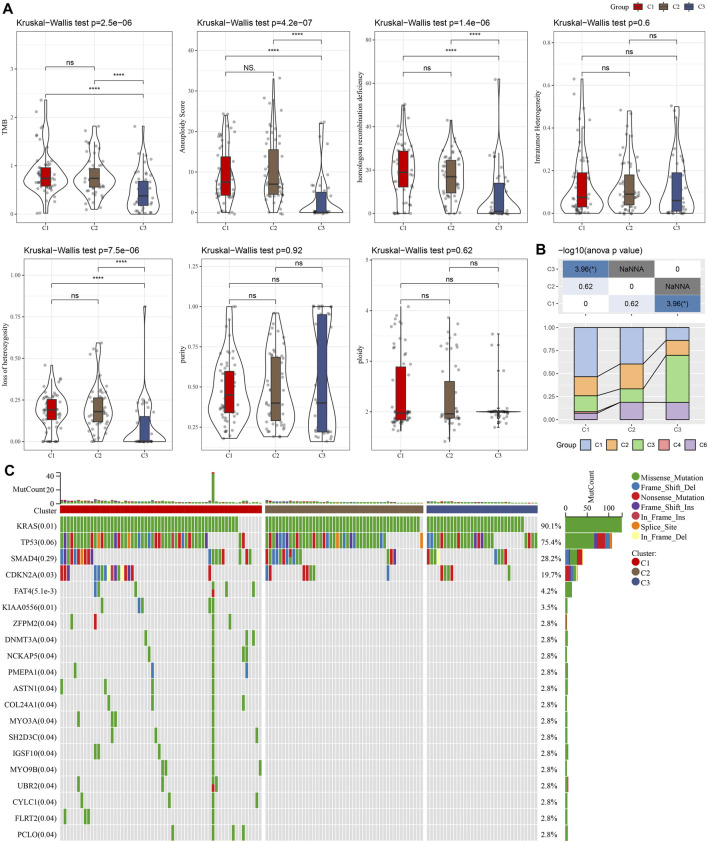
The comparison between our defined cluster with other existing subtypes. **(A)** The comparison of our defined clusters with the subtypes derived from the pan-cancer study. **(B)** The comparison of our defined clusters with immune signature-derived subtypes. **(C)** The comparison of somatic mutations in NKGs-derived subtypes in the TCGA cohort. *p < 0.05; **p < 0.01; ***p < 0.001; and ****p < 0.0001.

We compared defined three clusters with the molecular subtypes derived from a pan-cancer study and immune signatures (Thorsson et al., 2018). As shown in Figure 2A, the C1 cluster presented with a higher TMB, aneuploidy, homologous recombination defects, and loss of heterozygosity. Meanwhile, a significantly higher proportion of immune signature-derived C3 subtype in our defined C3 subtype was observed (Figure 2B). The immune signature-derived C3 subtype was characterized by the overexpression of TH17 and Th1 genes, a low to moderate proliferation rate of tumor cells, and lower levels of aneuploidy and overall somatic copy number alterations. Meanwhile, the immune signature-derived C3 subtype showed a better prognosis than other subtypes, which is consistent with our defined C3 cluster showing the best prognosis, as shown in Figure 1F. The gene mutations in each cluster were compared and the top 20 genes with a lower *p*-value were illustrated in Figure 2C. Most mutations were present in KRAS, TP53, and SMAD4, accounting for 75.3%, 28.2%, and 19.7%, respectively. It was noticed that the C1 cluster with a poor prognosis had more gene mutations.

### 3.3 Pathway characteristics among molecular subtypes

**FIGURE 3:**
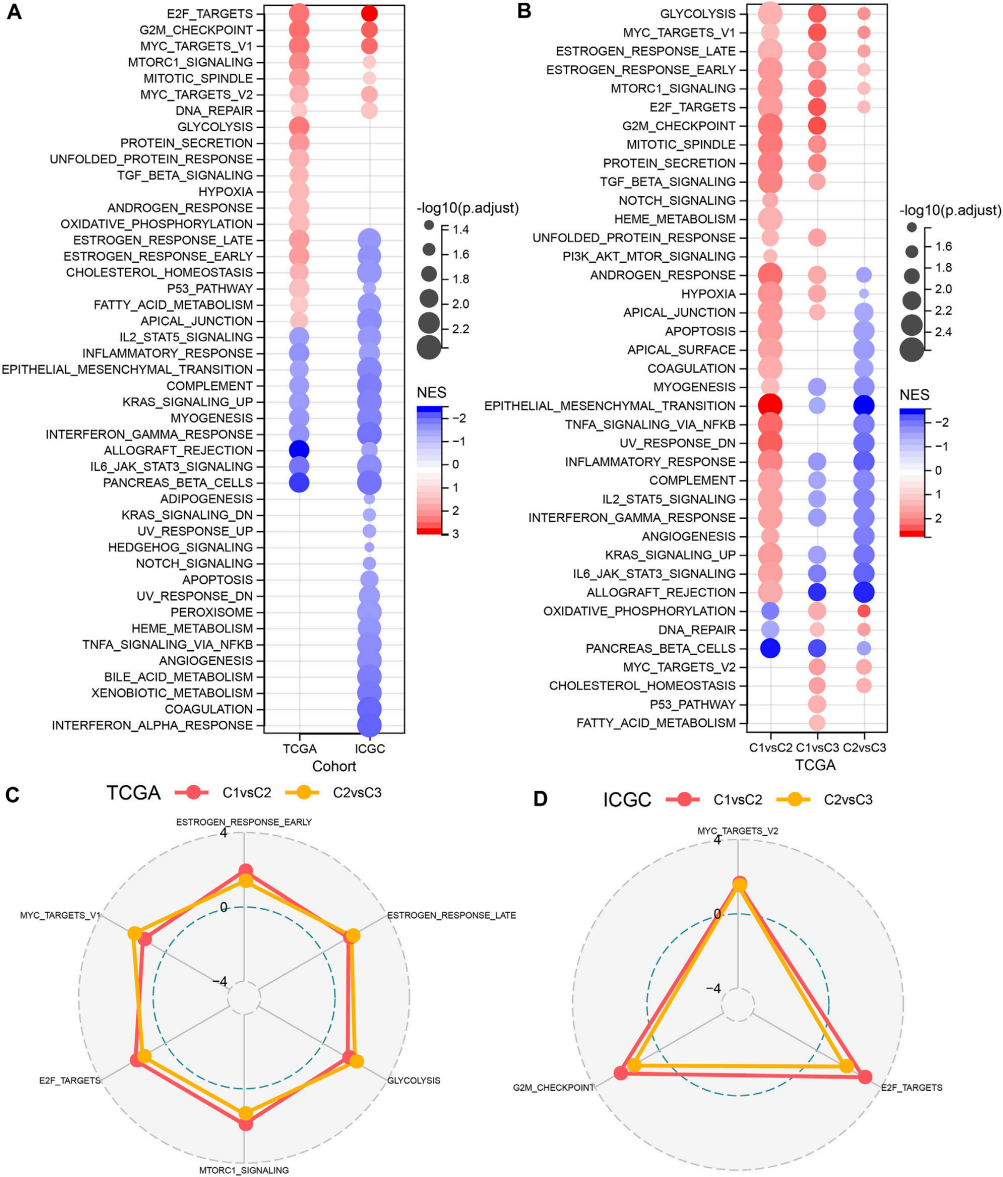
The comparison of pathways between molecular subtypes. **(A)** Bubble chart of GSEA analysis results of the TCGA cohort and the ICGC cohort. **(B)** Bubble chart of the GSEA analysis results of C1 vs. C3, C1 vs. C2, and C2 vs. C3 in the TCGA cohorts; **(C)** The radar chart of the C1vsC2, C2vsC3 coherent activation pathway in the TCGA cohort; **(D)** The radar plot of the C1vsC2, C2vsC3 coherent activation pathway in the ICGC cohort.

GSEA was performed to elucidate the pathway features in each cluster by using the Hallmark candidate gene sets. As shown in Figure 3A, the C1 cluster was significantly enriched in 38 pathways in the TCGA cohort. Generally, the activated pathways mainly included cell cycle-related pathways, such as E2F_TARGETS, G2M_CHECKPOINT, MYC_TARGETS_V1, whereas the inhibited pathways primarily contained INFLAMMATORY_RESPONSE, COMPLEMENT, and INTERFERON_GAMMA_RESPONSE. Similar results were also observed in the PACA-CA cohort. In addition, we compared the pathway characteristics between clusters (Figures 3B–D). It revealed that PC patients with the 3 subtype had activated immune pathways, such as cell cycle-related pathways, indicating that the 32 NKGs might play vital roles in the regulation of cell cycle and TME.

### 3.4 Immune signatures between molecular subtypes and differences in immunotherapy/chemotherapy/PCD

**FIGURE 4:**
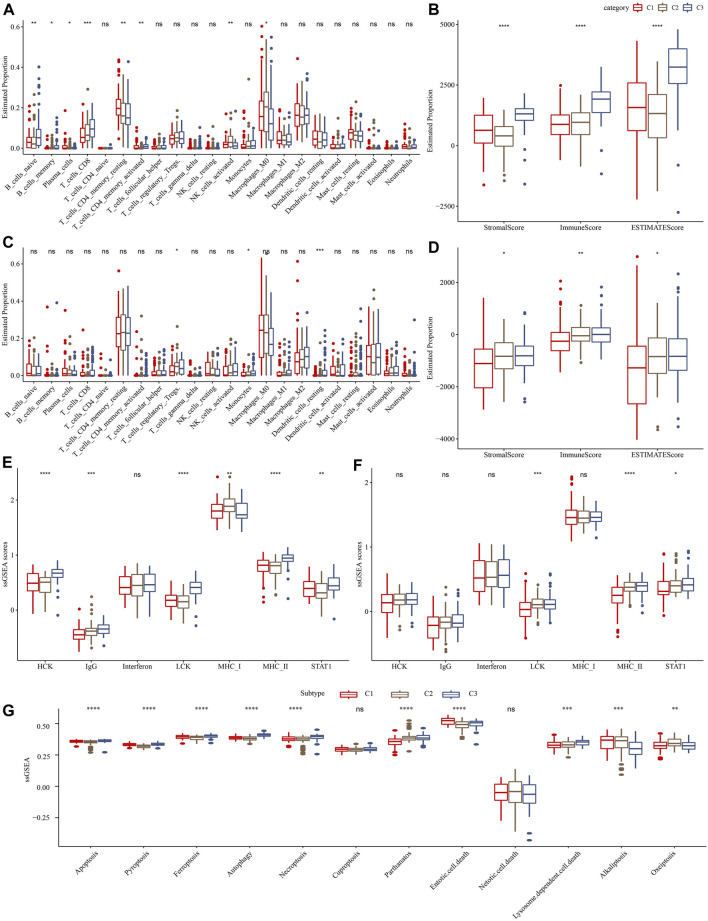
Comparison of immune infiltration and inflammation activity among three molecular subtypes. **(A)** and **(C)** represented the differences in the relative abundance of 22 immune cells among different molecular subtypes in the TCGA-PAAD and PACA-CA cohorts, respectively. **(B)** and **(D)** represented the comparison of the ESTIMETE results among clusters in the TCGA-PAAD and PACA-CA cohorts, respectively. **(E)** and **(F)** represented the differences in the inflammation activity among clusters in the TCGA-PAAD and PACA-CA cohorts, respectively. **(G)** The ssGSEA score differences of 12 programmed cell death patterns among 3 molecular subtypes.

Furthermore, we assessed the relative abundance of 22 immune cells in the TCGA-PAAD and PACA-CA cohorts using the CIBERSORT algorithm. As shown in Figures 4A, C, significant differences among three clusters were observed for several immune cell types, such as CD8+T cells and activated CD4^+^ memory T cells. Meanwhile, we observed a significantly higher immune score in the C3 cluster than in other clusters (Figures 4B, D), indicating that the C3 cluster had a higher immune infiltration. In addition, we investigated the 7 inflammation-related metagenes clusters in the three molecular subtypes. As a result, 6 of the 7 metagenes clusters were significantly differently expressed among subtypes, except for interferon (Figure 4E). Overall, the C1 cluster presented with a higher inflammation activity than other clusters. Meanwhile, we also observed a higher enrichment score of LCK and MHC-II, and STAT1 in the C1 cluster than the other two clusters in the PACA-CA cohort (Figure 4F). The ssGSEA analysis of 12 PCD patterns indicated that 10 PCD patterns had obviously differences among 3 subtypes, and in general, C1 or C3 subtype had higher ssGSEA scores (Figure 4G).

### 3.5 Immunotherapy response and drug sensitivity among clusters

**FIGURE 5:**
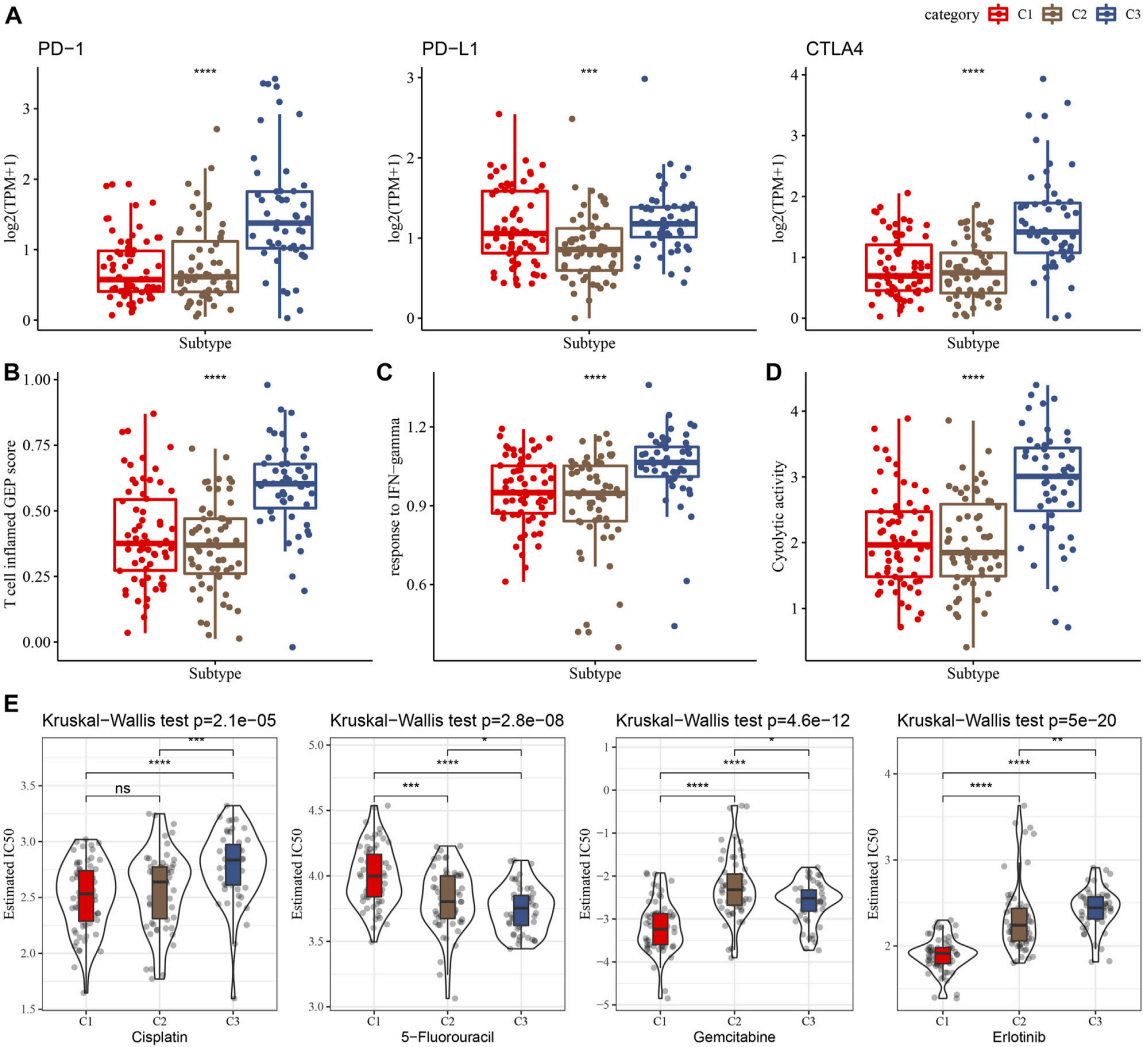
Differences in the immunotherapy response and drug sensitivity among clusters in the TCGA cohort. **(A)** Comparison of the ICGs among clusters. **(B–D)** Showing comparisons of the T cell inflamed GEP score, response to IFN-γ, and Cytolytic activity among clusters, respectively. **(E)** The box plots of the estimated IC_50_ for cisplatin, 5-Fluorouracil, gemcitabine and erlotinib in the three clusters.

Immunotherapy achieved favorable therapeutic effects in various cancers and immune checkpoint genes (ICG) play vital roles in these processes. Therefore, we evaluated the expression of ICGs among clusters and found an elevated expression of PD-1, PD-L1, and CTLA4 in the C3 cluster, as shown in Figure 5A. Meanwhile, we assessed the capability of clusters in predicting immunotherapy response using the T cell inflamed GEP score and observed a higher score in the C3 cluster than in other clusters (Figure 5B). INF-γ is a cytokine that plays a key role in immune regulation and anticancer immunity (Zhang et al., 2017), therefore, we calculated the ssGSEA score of the GOBP_RESPONSE_TO_INTERFERON_GAMMA gene set and found a significantly higher score of INF-γ response in the C3 cluster (Figure 5C). In addition, we also observed a higher CYT score in the C3 cluster than in other clusters (Figure 5D), which was used to reflect cytotoxic effects. Moreover, our data showed that the C1 cluster was more sensitive to cisplatin, gemcitabine, and erlotinib.

### 3.6 Establishment of a risk signature

**FIGURE 6:**
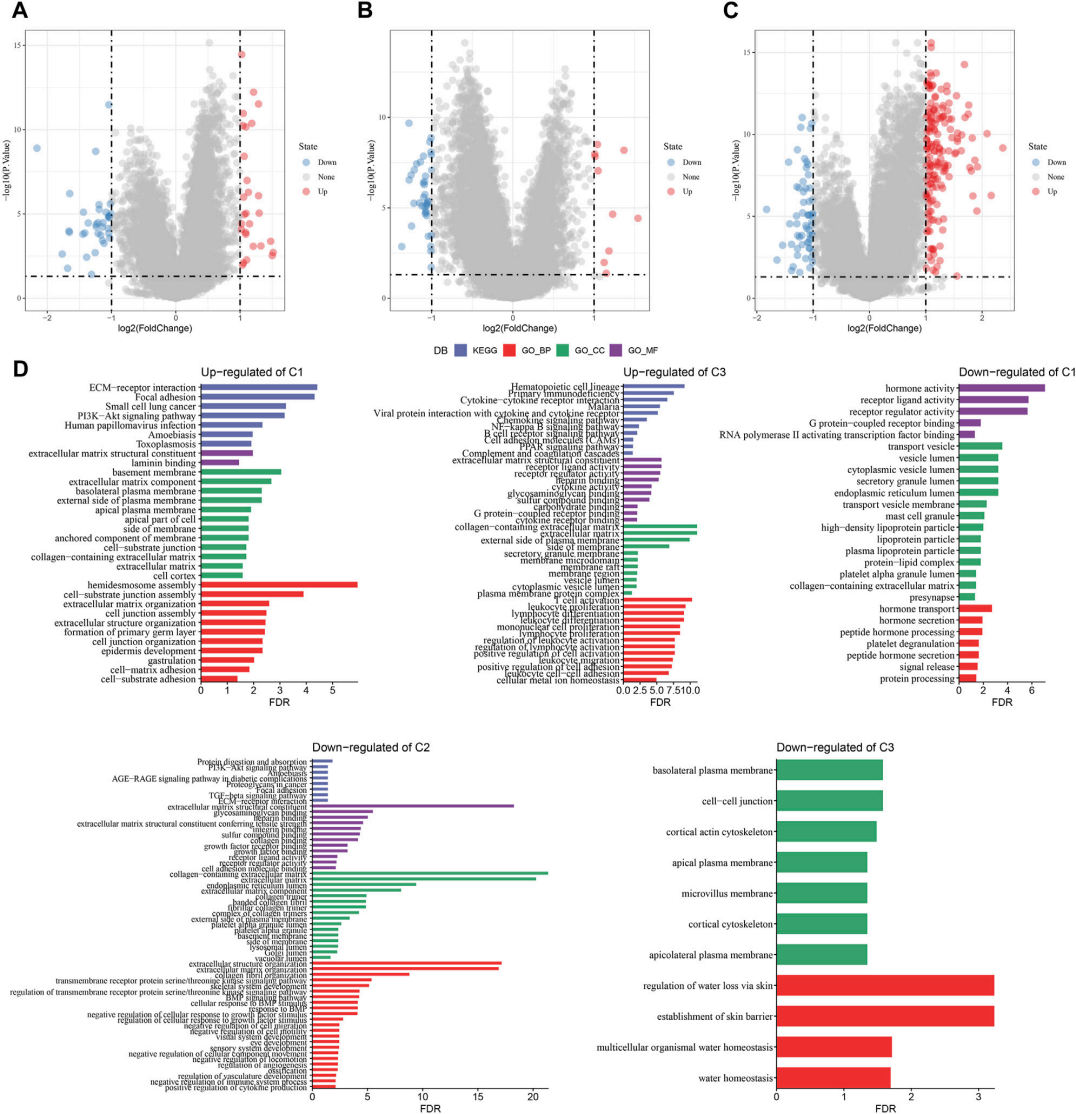
The identification of DEGs in each cluster. **(A–C)** Volcano plot of DEGs in the TCGA-PAAD cohort; **(D)** Functional enrichment analysis of DEGs of each cluster.

**FIGURE 7:**
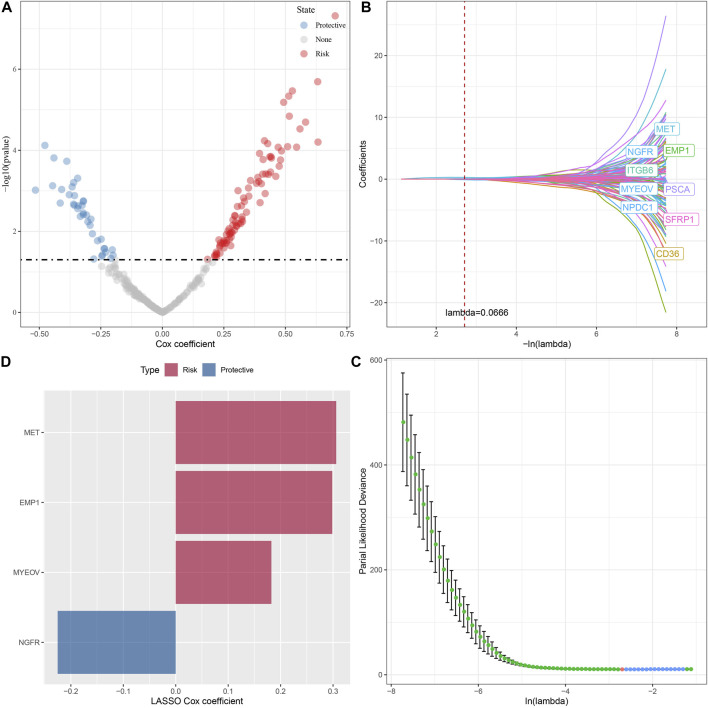
The identification of hub genes for the construction of the prognosis model, **(A)** A total of 122 promising candidates were identified from the DEGs; **(B)** The trajectory of each independent variable with lambda; **(C)** Confidence interval under lambda; **(D)** Distribution of LASSO coefficients of the Natural Killer Cell-related prognostic gene signature.

**FIGURE 8:**
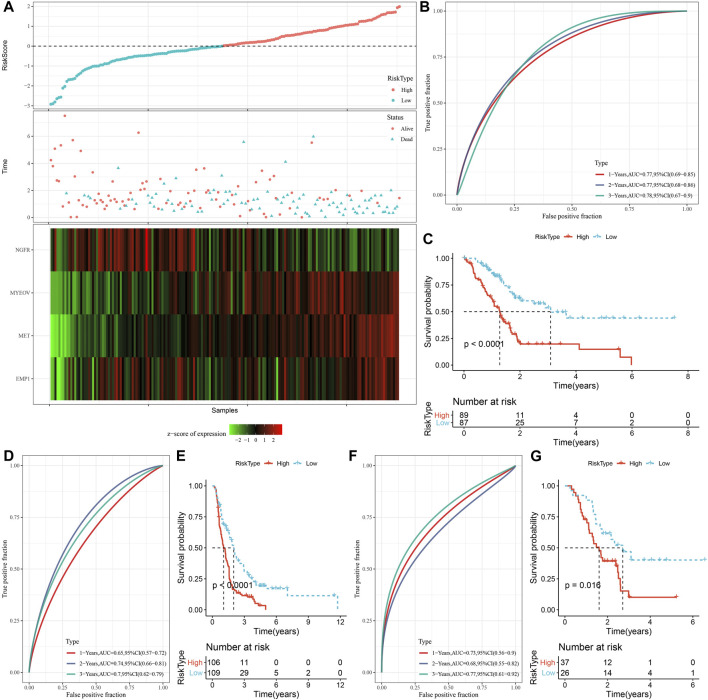
Construction and validation of the prognosis model for PC. **(A)** The risk scores of patients in the TCGA cohort. **(B)** The ROC results of the prognostic model in the TCGA cohort. **(C)** The survival analysis results of the prognostic model in the TCGA cohort. **(D, E)** ROC curve and KM survival curve of risk score in PACA-CA cohort; **(F, G)** ROC curve and KM survival curve of risk score in GSE57495 cohort.

A total of 294 DEGs among clusters were identified, as shown in Figures 6A–C. Enrichment analysis on the DEGs was performed and the results showed that the C3 cluster contained DEGs that were significantly associated with immune-related pathways (Figure 6D). Univariate COX analysis showed that 122 of the 293 DEGs were significantly associated with the prognosis of PC (*p* < 0.01), including 84 risk genes and 38 protective genes (Figure 7A). Subsequently, lasso COX regression was adopted to compress the gene number and found 9 candidate genes when lambda = 0.0666 (Figures 7B, C). Finally, four genes were identified after stepwise multivariate regression analysis on the 9 candidate genes and were used to construct a prognosis model (Figure 7D), RiskScore = +0.306*MET+0.299*EMP1-0.225*NGFR+0.182*MYEOV. The four gene expressions were negatively correlated with methylation level (Supplementary Figure S2). The risk score was calculated for each patient in the TCGA cohort and was used to divided the patient into the high and low group (Figure 8A). ROC analysis demonstrated a favorable predictive capability in forecasting the 1-, 3-, and 5-year survival rates (Figure 8B). Survival analysis showed a significantly difference in prognosis between the high and low groups (Figure 8C). In addition, we evaluated the robustness of the prognosis model in the PACA-CA and GSE57495 cohorts, which had similar results as the TCGA cohort (Figures 8D–G).

### 3.7 Differences in clinicopathological features and clusters between the high and low groups

**FIGURE 9:**
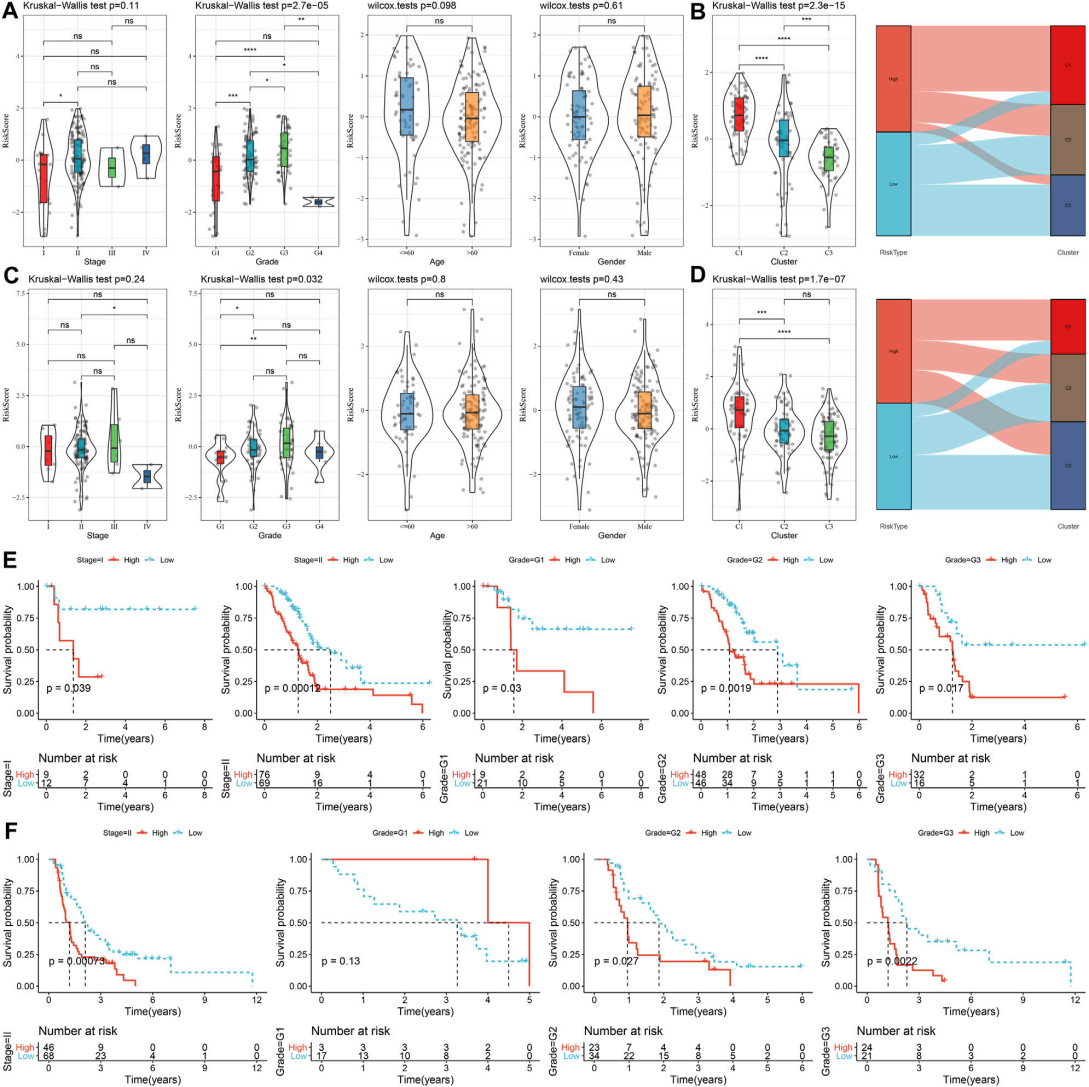
The distribution and predictive capability of the risk score in sub-population with distinct clinicopathological features. **(A)** and **(C)** Differences in risk score among different clinicopathological groups in the TCGA-PAAD and PACA-CA cohorts, respectively; **(B)** and **(D)** Difference in risk score among different molecular subtypes in the TCGA-PAAD and PACA-CA cohorts, respectively; **(E)** and **(F)** K-M curve of risk score-derived groups in different clinicopathological groups the TCGA-PAAD and PACA-CA cohorts, respectively.

The correlations between risk score and clinicopathological characteristics were analyzed in the TCGA and PACA-CA cohorts, and the results found significant associations between risk score and grade, but not stage, age, and gender (Figures 9A, C). Meanwhile, the risk score was significantly different among the three clusters, which manifested by a higher risk score in the C1 cluster and a lower risk score in the C3 cluster (Figures 9B, D). In addition, K-M curves showed that the risk score exhibited a favorable capability in the prognostic prediction of PC in sub-populations with specific clinicopathological features (Figures 9E, F).

### 3.8 Immune infiltration and pathway characteristics in different risk groups

**FIGURE 10:**
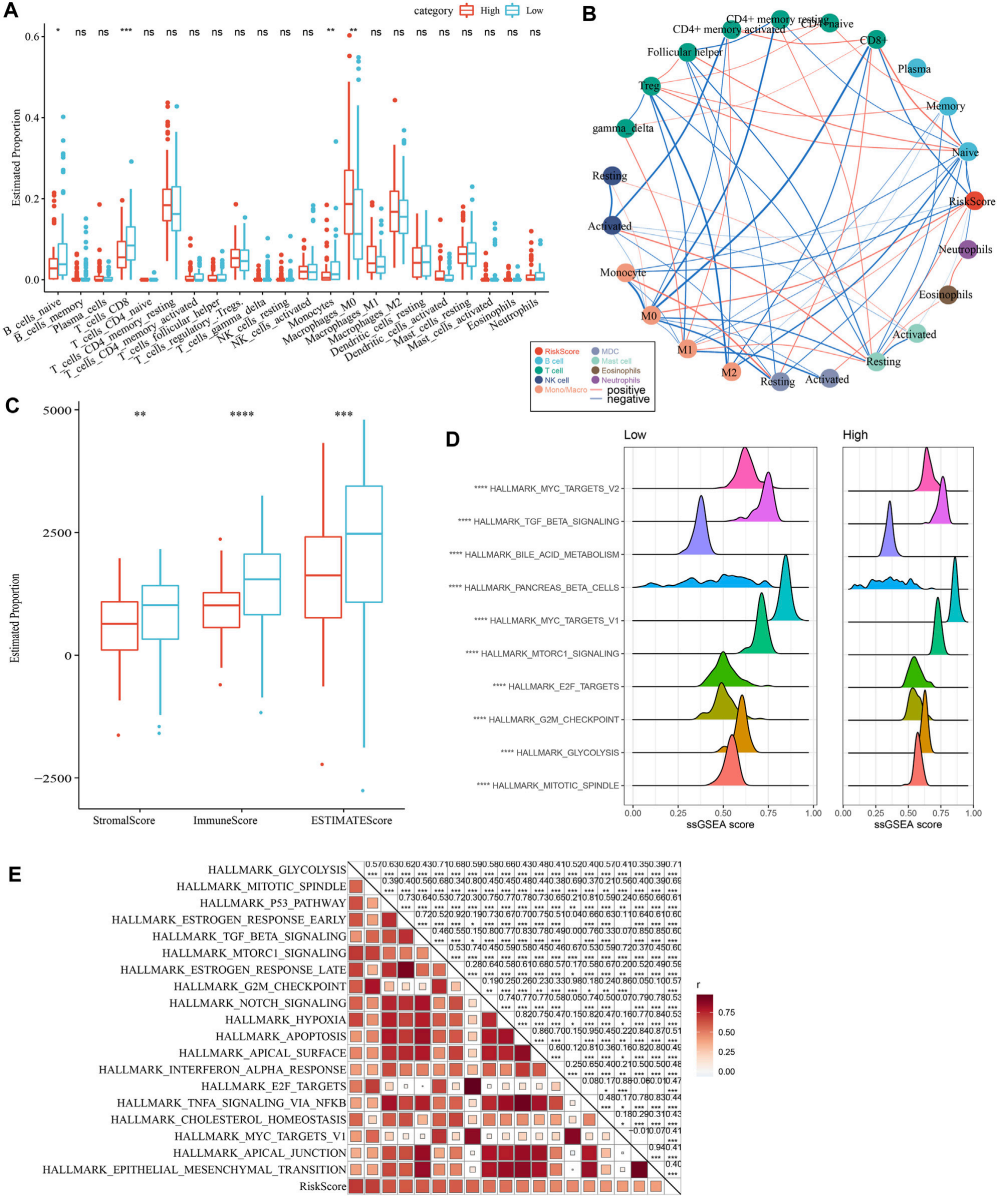
Comparison of immune infiltration and pathways between two risk groups. **(A)** Comparison of the proportion of immune cells in the TCGA cohort; **(B)** Correlation analysis between 22 immune cells and risk score in the TCGA cohort; **(C)** Comparison of the results of ESTIMATE between two risk groups; **(D)** The top 10 pathway with the most significant difference between the two risk groups. **(E)** Correlation analysis between pathways and risk score.

As shown in Figure 10A, we observed a significantly difference in the relative abundance of four immune cells, including naive B cells, CD8 T cells, monocytes, and M0 macrophages, between the high and low groups in the TCGA cohort. The correlations between risk score and immune cells were illustrated in Figure 10B. In addition, a higher immune score was observed in the low group than the high group, indicating a higher immune infiltration in the low group (Figure 10C). The ssGSEA scores on each pathway were calculated for individuals and were compared between two risk groups. The results demonstrated that the High group was significantly associated with cell cycle-related pathways (Figures 10D, E).

### 3.9 Immunotherapy response, chemotherapy sensitivity and PCD between two risk groups

**FIGURE 11:**
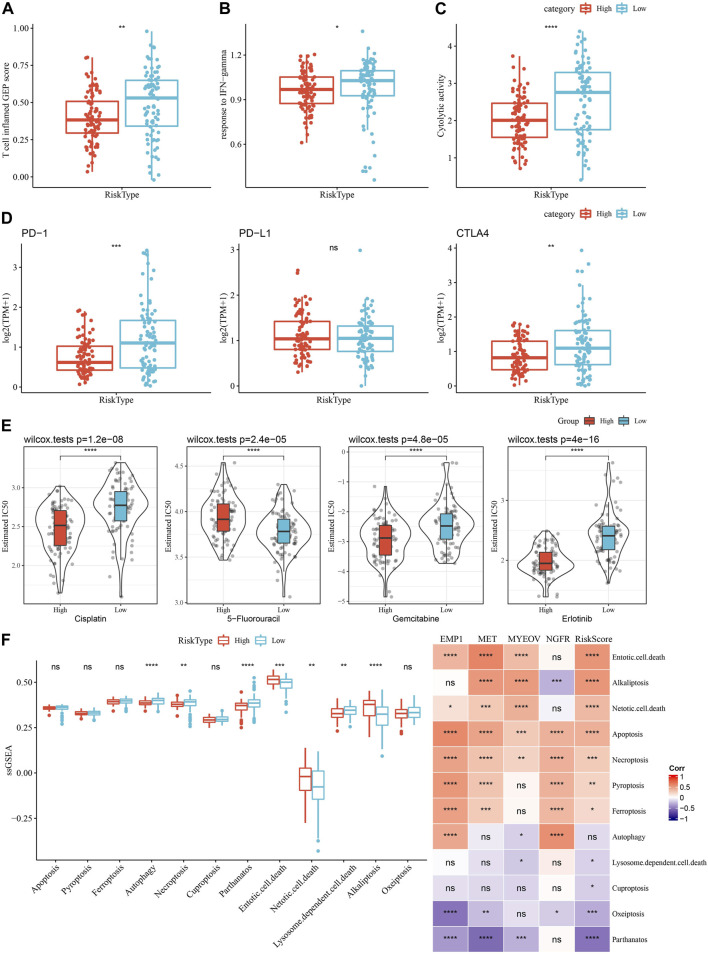
Comparison of the immunotherapy response and chemotherapy sensitivity between two risk groups in TCGA-PAAD cohort. **(A–C)** Represented the comparison of the T cell inflamed GEP score, response to IFN-γscore and cytolytic activity between the two risk groups, respectively. **(D)** Differences of expression ICGs between different groups; **(E)** The box plots of the estimated IC_50_ for cisplatin, 5-Fluorouracil, gemcitabine and erlotinib between the risk groups. **(F)** Left, the ssGSEA score differences of 12 programmed cell death patterns between high- and low-group. Right, the correlation analysis between 12 programmed cell death patterns and RiskScore.

As shown in Figure 11A, we observed a significantly higher T cell inflamed GEP score in the Low group as compared with those in the High group. Our data also revealed a significantly higher response to IFN-γ and cytolytic activity in the Low group, when compared with the high group (Figures 11B, C). In addition, we found elevated expression of PD-1 and CTLA4, but not PD-L1, in the low group (Figure 11D), suggesting potential differences in immunotherapy response between the two risk groups. Chemotherapy sensitivity in different risk groups was analyzed and found that the patients in the high group were more likely to be sensitive to gemcitabine, cisplatin, and erlotinib, as shown in Figure 11E. In addition, four of 12 PCD patterns had increased ssGSEA score in low group, while 3 PCD had higher ssGSEA score in high group (Figure 11F, left). Furthermore, we analyzed the correlation between RiskScore, four model genes and 12 PCD patterns, and there were different degrees of correlation with each other (Figure 11F, right).

### 3.10 Improvement of the prognostic model

**FIGURE 12:**
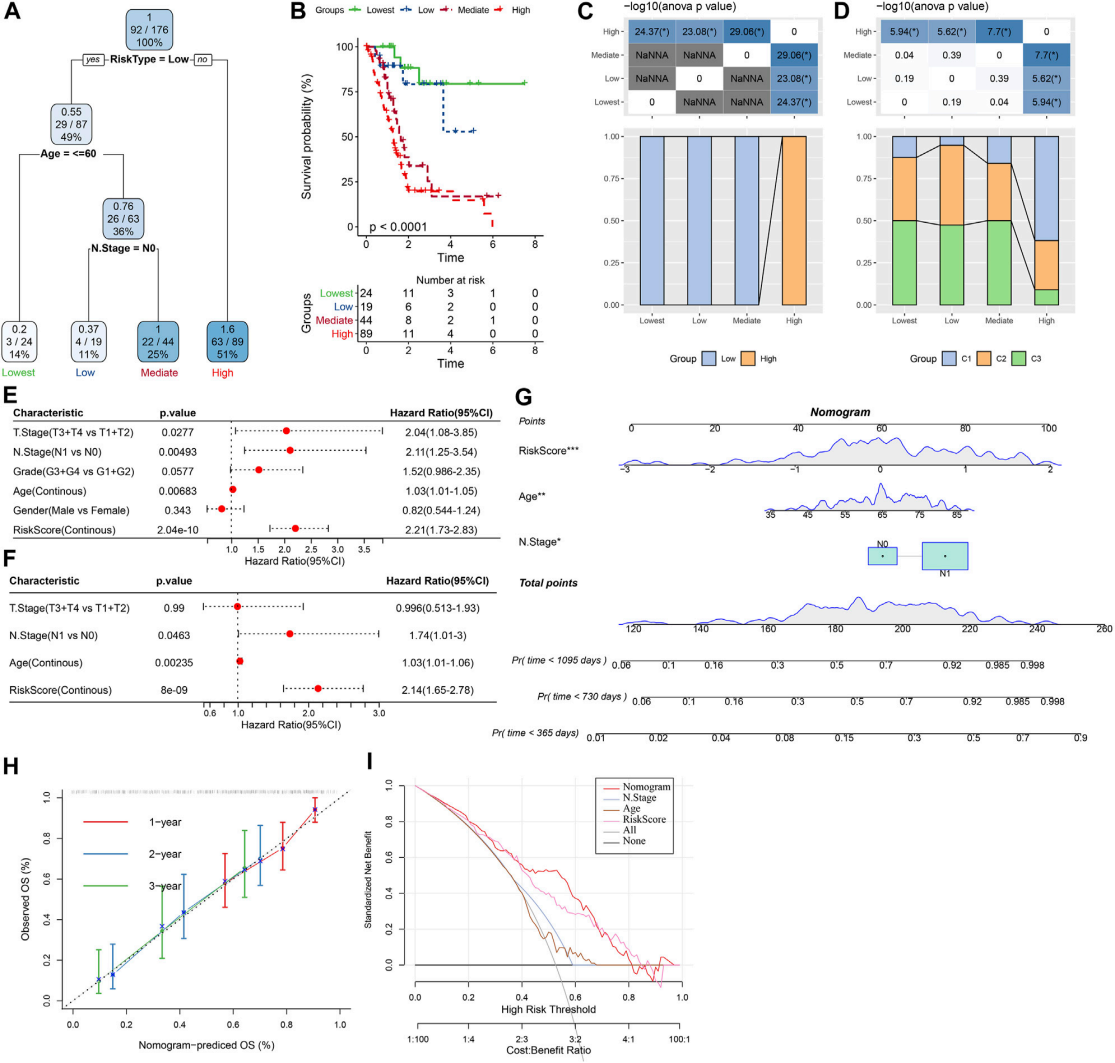
Construction of prognostic models of PC using the risk score and clinicopathological features. **(A)** A decision tree model generated four risk groups based on age, N stage and the risk score. **(B)** Survival analysis of the decision tree-derived risk groups showing distinct prognoses among the groups. **(C, D)** showed the correlations between risk score-derived groups and decision tree-derived groups. **(E)** and **(F)** Univariate and multivariate Cox analysis of risk score and clinicopathological characteristics. **(G)** The nomogram model consists of age, N stage, and the risk score; **(H)** 1-, 2-, and 3-year calibration curves of the established nomogram; **(I)** The decision curves showing the capacity for survival prediction.

As shown in Figure 12A, a decision tree was constructed based on the risk score and clinicopathological features and generated four groups (Lowest, Low, Mediate, High) using three parameters (risk score, N stage, age). Survival analysis demonstrated significant differences in prognosis among the four groups (Figure 12B, *p* < 0.001). The correlations between the decision tree-derived groups and risk groups were illustrated in Figures 12C, D. Univariate regression analysis showed that T stage, N stage, age, and risk score was associated with the prognosis of PC, and three of them (N stage, age, and risk score) were identified as independent risk factors *via* multivariate regression analysis (Figures 12E, F). Therefore, a nomogram was built using the three factors (Figure 12G). It was observed that the predicted values were close to the observed values in terms of the 1-, 2, and 3-year OS (Figure 12H), indicating that the nomogram had good prediction performance. In addition, a decision curve was used to evaluate the reliability of the model, and it was observed that the risk signature and nomogram model had a higher standardized net benefit as compared with other clinicopathological features (Figure 12I).

## 4 Discussion

Tumor immunotherapy has brought hope for cancer treatment, and more and more studies have shown that innate immune cells, including NK cells, have unique advantages in anti-tumor immunotherapy. However, most of the current research focuses on adaptive immune cells, and the role of innate immune cells has not been paid enough attention. Studies have shown that the abundance of tumor infiltrating NK cells is closely related to the prognosis of patients with various solid tumors (Villegas et al., 2002; Cursons et al., 2019; Meng et al., 2019). The prognostic model based on NKG has the potential ability to predict prognosis and immunotherapy response (Cursons et al., 2019). Meanwhile, a novel human NK cell-based immunotherapy was developed and showed efficacy in human metastatic PC models (Teng et al., 2022). Inspired by these findings, we attempted to investigate the molecular subtypes of PC based on prognosis-related NKGs using transcriptomic data in this study. Distinct differences in prognosis, immunotherapy response, and drug sensitivity among subtypes were observed, indicating the crucial role of NK cells in the progression and treatment of PC. Functional enrichment analysis showed that NKGs involved in activated immune pathways, such as cell cycle-related pathways, indicating that the those NKGs might play vital roles in the regulation of cell cycle and TME. Furthermore, we developed a novel prognostic prediction signature based on DEGs that were found among NKGs-derived molecular subtypes of PC, which exerts a favorable capability of prognostic prediction.

Herein, we identified 32 prognosis-related NKGs in PC, including 12 protective genes and 20 risk genes, and the expression of most of these genes was significantly correlated. A number of studies had proposed potential roles of these NKGs in PC. For instance, the tumor necrosis factor ligand superfamily member 10 (TNFSF10), also known as TRAIL, encodes a cytokine that belongs to the tumor necrosis factor (TNF) ligand family, it preferentially induces apoptosis in transformed and tumor cells and was proposed as a prognostic indicator of PC (Wang et al., 2021; Wang et al., 2022). As a well-known driver gene, KRAS frequently mutated in PC patients (Waters and Der, 2018), our data revealed that KRAS was the most mutated gene in the TCGA-PAAD cohort. KRAS gene mutations has been reported to be involved in the invasion and metastasis of tumor cells, as well as chemoresistance (Mueller et al., 2018; Buscail et al., 2020). It was found that TMB was associated with the sensitivity of immunotherapy response and was more effective than ICG expression in screening patients suitable for immunotherapy (Choucair et al., 2020). This finding may result from the enrichment of immune cells due to the elevated production of “non-self” neoantigen under high TMB (Schumacher and Schreiber, 2015). In addition, it was observed that the phosphatidylinositol 4,5-bisphosphate 3-kinase catalytic subunit beta isoform (PIK3CB) was involved in metastasis of PC cells (Qu et al., 2021). Therefore, further investigation on these prognostic NKGs and their mutations might provide clues for the development of novel treatment of PC.

Three stable clusters with distinct differences in prognosis were generated based on the prognostic NKGs, and GSEA results found significant differences in cell cycle pathways and immunity-related pathways among clusters. Therefore, the inferior prognosis of patients in the C1 cluster may be partly attributed to the disturbance of cell cycle regulation, which is closely related to tumor proliferation and progression (Tang et al., 2020). Meanwhile, these data indicated that the prognostic NKGs used for molecular typing play important roles in the cell cycle process and tumor immune microenvironment. For example, Rac1 plays an important role in regulating cell function, and its activation affects cell morphology (Etienne-Manneville and Hall, 2002), cell cycle and gene expression (Yoshida et al., 2010), survival and apoptosis (Liang et al., 2021). Tyrosine kinase FYN was reported to be associated with mediating mitogenic signals and involved in regulating cell cycle and proliferation (Zheng et al., 2017). Besides, we observed significant differences in immune cells infiltration among NKG-derived clusters. The C1 cluster was characterized as so-called “cold tumor” since it presented with a lower immune cell infiltration. The tumor-infiltrating immune cells participated in tumor development and influence prognosis (Barnes and Amir, 2017), and anti-tumor activity of “cold tumor” is decreased because low immune cell infiltration could increase tumor cell escape from immune surveillance and contribute to tumor progression (Bonaventura et al., 2019). These finding may partly contribute to the significant reduction in survival of the C1 and C2 clusters. Meanwhile, a lower stromal score was observed in the C1 and C2 clusters, which was suggested to be associated with a poor OS of osteosarcoma (Alves et al., 2019).

Since GSEA revealed significant inhibition of inflammatory response among clusters, we further evaluated the relationships between NKG-derived clusters and inflammatory activities by analyzing inflammatory-related metagenes. Notably, significant differences in hematopoietic cell kinase (HCK), IgG, MHC-II, src-family kinases p56 (LCK), MHC-I, and were observed among clusters. HCK plays a pivotal role in innate immunity and was overexpressed in various cancers. It could regulate the phagocytosis of neutrophils and macrophages (Roseweir et al., 2019), as well as immune cell infiltration in the TME (Cheng et al., 2022). LCK is critical for proximal T-cell antigen receptor (TCR) signal transduction and is involved in the earliest steps of TCR-mediated T-cell activation (Salmond et al., 2009). MHC-I and MHC-II are two pivotal molecules presenting with the function of antigenpresentation, and their loss of expression would make tumor cells escape T-cell killing (Garrido and Aptsiauri, 2019). Therefore, a lower level of these inflammatory-related metagenes may partly account for the immunosuppressive microenvironment in the C1 and C2 clusters.

Discrepancy between inflammatory activities and immune cell infiltration among clusters prompted us to explore the immunotherapy response. It was suggested that ICG expression partly contribute to the success of immune checkpoint blockade therapy. Herein, we revealed significant differences in ICG expression among clusters, indicating potential differences in the response to immunotherapy among clusters. In addition, a T cell-inflamed gene expression profile (GEP) was found to be effective in predicting response to anti-PD-1-directed therapy (Ayers et al., 2017). Our data showed that the C3 cluster had a significantly higher T cell-inflamed GEP score, indicating that PC patients in the C3 cluster might be more sensitive to anti-PD-1 therapy. Cytokine IFN-γ plays a key role in anticancer immunity and immune regulation, and the C3 cluster presented with a higher elevated expression of the gene set that responds to IFN-γ. Moreover, the cytolytic activity score (CYT) has been considered as a useful tool to evaluate anti-tumor immunity. It has been revealed that high CYT was associated with better prognosis of colorectal cancer, which could be explained by increased immunity and cytolytic activity of T cells and M1 macrophages (Narayanan et al., 2018). In this study, elevated cytolytic activity was observed in the C3 cluster. Moreover, our data also revealed the differences in chemotherapeutic drug sensitivity among clusters. The clusters derived from NKG have significant differences in immunotherapy and chemotherapy responses, which has potential value to guide individualized treatment strategies.

On the basis of NKG-derived clusters, we established a novel prognostic signature using the DEGs found among clusters. This prognostic signature has satisfactory prognostic performance and has shown good predictive power in immunotherapy response and chemotherapeutic drug sensitivity. Despite the promising findings obtained, several limitations in this study should be acknowledged. First, due to the high heterogeneity of the tumor immune microenvironment, the prognosis-predicting ability of NKG-derived molecular subtypes and subsequent prognostic models was limited. Second, analysis of NK cell characteristics based on single cell sequencing will help to further understand its role in PC. Finally, further study is required to investigate the underlying mechanism of the genes in the risk signature and PC patients’ outcomes.

## 5 Conclusion

In conclusion, we established three molecular clusters of PC using 32 prognosis-related NKGs and revealed differences in clinicopathological and genomic features, pathways, immunotherapy response, and drug sensitivity among clusters. Furthermore, a prognostic signature with robust prognosis-predicting ability was built and validated. The NKG-derived molecular clusters and prognostic signature might serve as a useful tool for assisting in the decision of individualized treatment and the selection of suitable individuals for chemotherapy.

## Data Availability

The datasets presented in this study can be found in online repositories. The names of the repository/repositories and accession number(s) can be found in the article/Supplementary Material.

## Supplementary material

SUPPLEMENTARY FIGURE S1 Working flow chart

SUPPLEMENTARY FIGURE S2 The correlation of expression and Methylation level in 4 genes

## Abbreviation

NK, Natural killer; NKGs, NK cell-related genes; PC, pancreatic cancer; TME, tumor microenvironment; TCGA, The Cancer Genome Atlas Program; GEO, Gene Expression Omnibus; PAM, partition around medoids; DEGs, differently expressed genes; FDR, false discovery rate; FC, fold change; LASSO, least absolute shrinkage and selection operator; ROC, receiver operating characteristic; MSigDB, Molecular Signatures Database; IC_50_, half-maximal inhibitory concentration; DCA, decision curve analysis; TMB, tumor mutation burden; ICG, immune checkpoint genes; TNFSF10, tumor necrosis factor ligand superfamily member 10; TNF, tumor necrosis factor; PIK3CB, phosphatidylinositol 4,5-bisphosphate 3-kinase catalytic subunit beta isoform; HCK, hematopoietic cell kinase; LCK, src-family kinases p56; TCR, T-cell antigen receptor; GEP, gene expression profile; CYT, cytolytic activity score.
