# Supplementary figures and images for: A novel natural killer cell-related signatures to predict prognosis and chemotherapy response of pancreatic cancer patients
Source: Front Genet. 2023 Mar 23;14:1100020. doi: 10.3389/fgene.2023.1100020 (PMC10076548; doi:10.3389/fgene.2023.1100020)

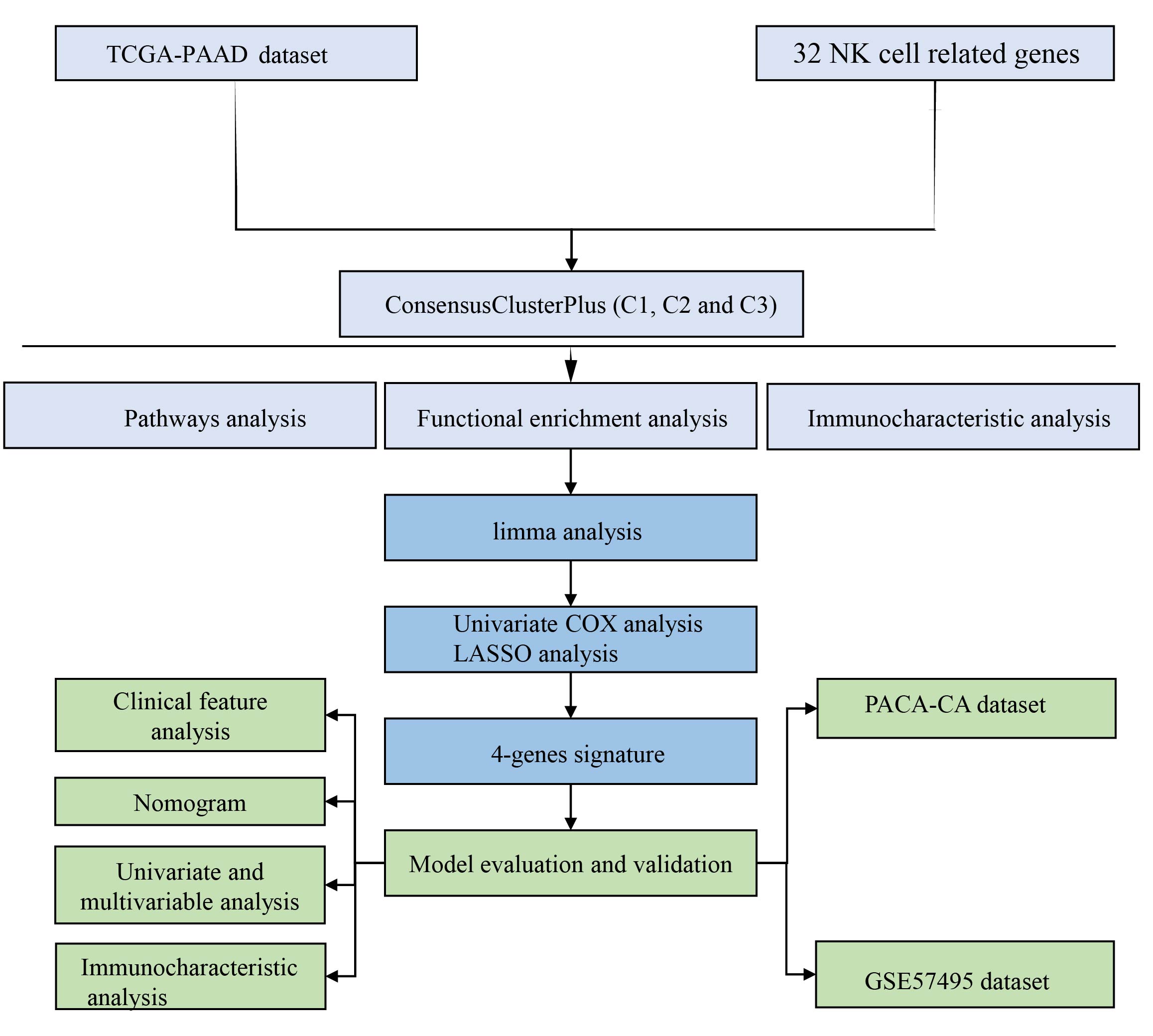

Supplement: Supplementary file 1 [file Image1.JPEG]

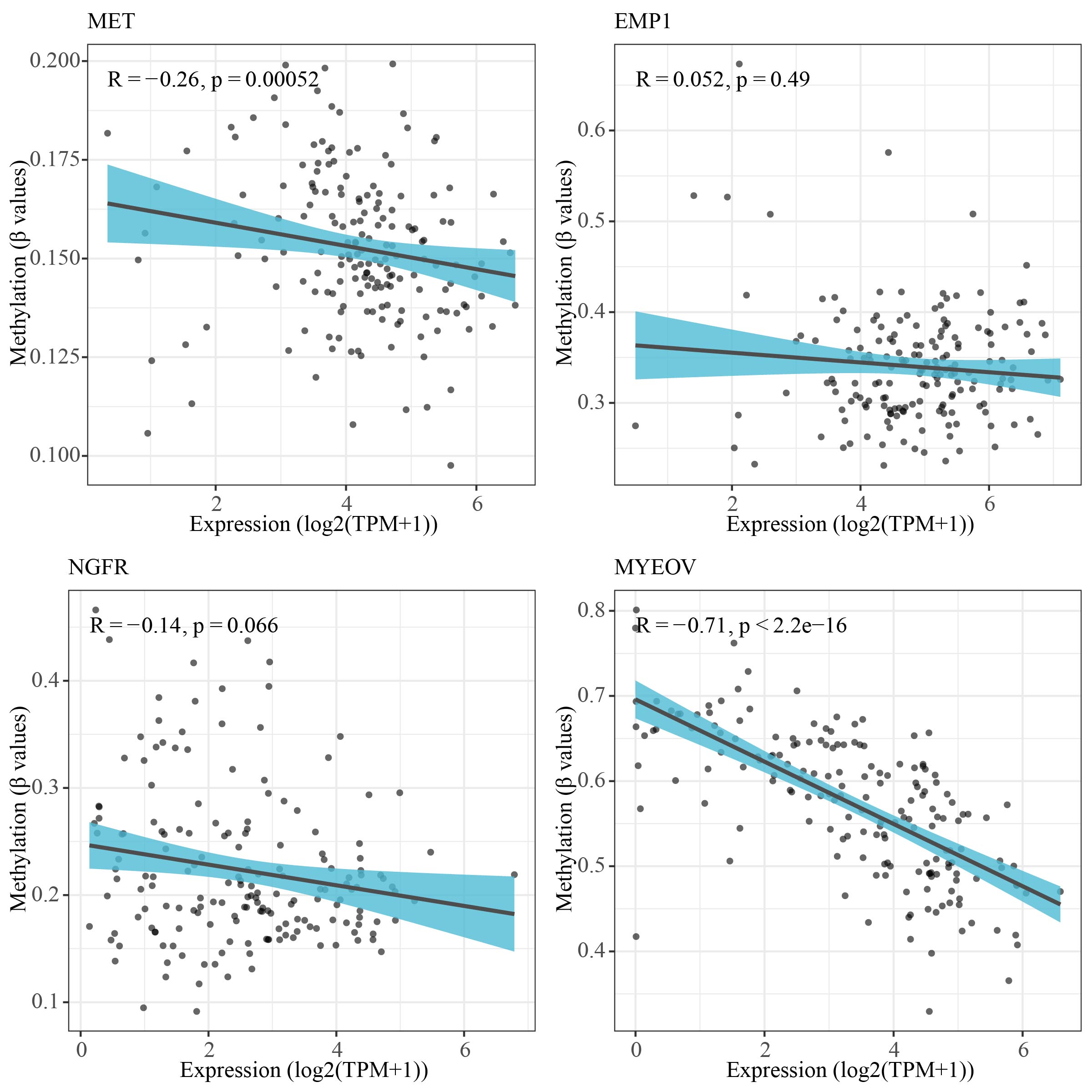

Supplement: Supplementary file 2 [file Image2.JPEG]
