# Supplementary material for: A novel natural killer cell-related signatures to predict prognosis and chemotherapy response of pancreatic cancer patients
Source: Front Genet. 2023 Mar 23;14:1100020. doi: 10.3389/fgene.2023.1100020 (PMC10076548; doi:10.3389/fgene.2023.1100020)
Supplement: Supplementary file 3 [file DataSheet1.docx]

Data analyzed in this study please see:

https://www.jianguoyun.com/p/DechFWYQ1Z6UCxixw-YEIAA
